# Phylogenetic Analysis of a ‘Jewel Orchid’ Genus *Goodyera* (Orchidaceae) Based on DNA Sequence Data from Nuclear and Plastid Regions

**DOI:** 10.1371/journal.pone.0150366

**Published:** 2016-02-29

**Authors:** Chao Hu, Huaizhen Tian, Hongqing Li, Aiqun Hu, Fuwu Xing, Avishek Bhattacharjee, Tianchuan Hsu, Pankaj Kumar, Shihwen Chung

**Affiliations:** 1 School of Life Sciences, East China Normal University, Shanghai, China; 2 Shanghai Key Laboratory of Plant Functional Genomics and Resources, Shanghai Chenshan Botanical Garden, Shanghai, China/Shanghai Chenshan Plant Science Research Center, Chinese Academy of Sciences, Shanghai, China; 3 School of Biological Sciences, The University of Hong Kong, Pokfulam Road, Hong Kong, China; 4 South China Botanical Garden, Chinese Academy of Sciences, Guangzhou, China; 5 Central National Herbarium, Botanical Survey of India, Botanic Garden, Howrah, India; 6 Institute of Molecular & Cellular Biology, National Tsing Hua University, Hsinchu, Taiwan; 7 Orchid Conservation Section, Flora Conservation Department, Kadoorie Farm & Botanic Garden, Lam Kam Road, Lam Tsuen, Tai Po, New Territories, Hong Kong, China; 8 Division of Forest Biology, Taiwan Forestry Research Institute, Taipei, Taiwan; National Cheng-Kung University, TAIWAN

## Abstract

A molecular phylogeny of Asiatic species of *Goodyera* (Orchidaceae, Cranichideae, Goodyerinae) based on the nuclear ribosomal internal transcribed spacer (ITS) region and two chloroplast loci (*matK* and *trnL-F*) was presented. Thirty-five species represented by 132 samples of *Goodyera* were analyzed, along with other 27 genera/48 species, using *Pterostylis longifolia* and *Chloraea gaudichaudii* as outgroups. Bayesian inference, maximum parsimony and maximum likelihood methods were used to reveal the intrageneric relationships of *Goodyera* and its intergeneric relationships to related genera. The results indicate that: 1) *Goodyera* is not monophyletic; 2) *Goodyera* could be divided into four sections, viz., *Goodyera*, *Otosepalum*, *Reticulum* and a new section; 3) sect. *Reticulum* can be further divided into two subsections, viz., *Reticulum* and *Foliosum*, whereas sect. *Goodyera* can in turn be divided into subsections *Goodyera* and a new subsection.

## Introduction

The genus *Goodyera* R. Br. (subtribe Goodyerinae; tribe Cranichideae; subfamily Orchidoideae) comprises *ca*. 40 species [1–4] and is widely distributed, including Asia, northeast Australia, Europe, South Africa, Madagascar, North America (including Mexico) and the southwestern Pacific islands [4]. Thirty-three species of *Goodyera* are recognized in China (with 12 endemics), showing widespread distribution mainly in south and southeastern China [4–9]. *Goodyera* is a genus of mainly terrestrial (rarely epiphytic) orchid which grows in shade on mossy rocks or along moist tracks of perennial mountain stream banks; it is characterized by the creeping rhizome, upper surface of leaves often with white or golden markings and veins, saccate labellum (glabrous or not internally), two sectile pollinia attached to a viscidium and a single stigmatic lobe [3]. Due to the remarkable markings on the leaves some taxa of this genus are known in horticulture as ‘jewel orchids’. The markings, as well as the overall colouration of the leaves of this genus, vary considerably depending on habitat conditions, and hence can be cause of confusion in field identification.

Schlechter [10] divided *Goodyera* into two sections: *Otosepalum* (lateral sepals reflexed) and *Eu-Goodyera* (lateral sepals normally spreading), his treatment was followed by Seidenfaden [11–12] and Pearce & Cribb [13]. However, some other scholars did not adopt Schlechter’s system because of the difficulty in defining the exact orientation of the lateral sepals. Lang [14] and Chen et al. [4] treated the markings on the leaves and whether the leaves are rosulate or not as important characters in distinguishing groups of species. Tian [15] did taxonomic studies of *Goodyera* in China based on morphological data where she also disagreed with Schlechter’s stand on section division, and preferred the lip sac hairy or not as more prominent feature than the orientiation of lateral sepals. However, most classifications of *Goodyera* have been based on morphological attributes.

The advent of molecular techniques has dramatically advanced our understanding of the phylogenetic relationships in family Orchidaceae. The internal transcribed spacer (ITS) region of nrDNA possesses moderate interspecific variation and has been the primary source of characters for phylogenetic analysis at lower taxonomic levels [16]. In the previous systematic studies based on molecular data, Shin et al. [17] conducted a phylogenetic analysis of five *Goodyera* species from Korea based on the ITS region which indicated that *Goodyera* was monophyletic, and the ITS sequences of *G*. *schlechtendaliana* Rchb. f. and *G*. *repens* (L.) R. Br. were identical to one another. The achievements were limited because of limited sampling and single DNA marker. Chung [18] investigated the systematics of *Goodyera* mainly based on taxa reported from Taiwan and concluded that the genus was monophyletic according to morphological and cytological data as well as ITS sequences. He divided *Goodyera* into three sections (including the two sections proposed by Schlechter [10] and a new section, *Reticulum* S. W. Chung & C. H. Ou), which were further subdivided into seven subsections (Table 1). But in his research, only one single ITS marker was used and all those new sections and subsections (Table 1) that he proposed without Latin diagnoses, which turned them invalid according to the Melbourne Code [19].

**Table 1:** Infrageneric Classifications of *Goodyera*.

Schlechter [10]	Chung [18]
**Sect. *Otosepalum* Schltr.**	**Sect. *Otosepalum* Schltr.**
*Goodyera papuana* Ridl.	**Subsect. *Otosepalum* Schltr.**
*Goodyera erythrodoies* Schltr.	*Goodyera carnea* (Bl.) Schltr.
*Goodyera angustifolia* Schltr.	*Goodyera erythrodoides* Schltr.
*Goodyera branchiorrhynchos* Schltr.	*Goodyera fumata* Thwaites
	*Goodyera glauca* Sm.
	*Goodyera grandis* Bl.
	*Goodyera maurevertii* Bl.
	*Goodyera polygonoides* Schltr.
	*Goodyera vitiensis* (Williams) Kores
	*Goodyera viridiflora* Bl
	**Subsect. *Procerum* S. W. Chung & C. H. Ou**
	*Goodyera procera* (Ker-Gawl.) Hook
**Sect. *Eu-Goodyera* Schltr.**	**Sect. *Goodyera* Schltr.**
*Goodyera lamprotaenia* Schltr.	**Subsect. *Goodyera* Schltr.**
*Goodyera stenotapetala* Schltr.	*Goodyera beccarii* Schltr.
*Goodyera venusta* Schltr.	*Goodyera bilamellata* Hayata
	*Goodyera bomiensis* Lang
	*Goodyera brachystegia* Hand.-Mazz.
	*Goodyera daibuzanensis* Yamamoto
	*Goodyera gemmata* Sm.
	*Goodyera kwangtungensis* Tso
	*Goodyera nantoensis* Hayata
	*Goodyera oblongifolia* Raf.
	*Goodyera pubescen* (Willd.) R.Br.
	*Goodyera repens*
	*Goodyera schlechtendaliana*
	*Goodyera secundiflora* Lindl.
	*Goodyera wolongensis* Lang
	*Goodyera wuana* Tang & Wang
	*Goodyera vittata* Benth. ex Hook.
	**Subsect. *Recurvum* S. W. Chung & C. H. Ou**
	*Goodyera recurve* Lindl.
	*Goodyera nankoensis* Fukuy.
	**Sect. *Reticulum* S. W. Chung & C. H. Ou**
	**Subsect. *Reticulum* S. W. Chung & C. H. Ou**
	*Goodyera alveolatus* Pradhan
	*Goodyera boninensis* Nakai
	*Goodyera colorata* (Bl.) Bl.
	*Goodyera hemsleyana* King & Pantling
	*Goodyera hispida* Lindl.
	*Goodyera lamprotaenia* Schltr.
	*Goodyera major* Ames & Correll
	*Goodyera pusilla* Bl.
	*Goodyera reticulata* (Bl.) Bl.
	*Goodyera ustulata* Carr
	**Subsect. *Foliosum* S. W. Chung & C. H. Ou**
	*Goodyera bifida* (Bl.) Bl.
	*Goodyera foliosa* (Lindl.) Benth. ex C. B. Clarke
	*Goodyera fusca* (Lindl.) Hook. f.
	*Goodyera velutina* Maxim.
	*Goodyera robusta* Hook. f.
	**Subsect. *Biflora* S. W. Chung & C. H. Ou**
	*Goodyera biflora* (Lindl.) Hook. f.

Juswara [20] utilized ITS and two chloroplast markers *trnL-F* and *rpl16* sequences to study the phylogyne of Goodyerinae. In her study, *Goodyera* was polyphyletic, 11 *Goodyera* species were split into two subclades each cluster with other genera in Goodyerinae. She adopted Schlechter’s classification and no further subsectional treatment was given.

As previous molecular systematics of *Goodyera* were largely based on samples from Tropical area [18, 20] or utilized a single DNA marker (ITS) [17, 18], the systematics of *Goodyera* is still unclear. Likewise, in other studies [21–30] only a few species of *Goodyera* have been included, which has not unable to resolve the phylogeny of *Goodyera* as a whole. The 33 species of *Goodyera* present in China represent *ca*. 83% of the total of 40 spp. in the genus worldwide, so the phylogenetic study on Chinese members of the genus is of high value in building up a global phylogenetic framework of *Goodyera*. Based on previous research [15, 17, 18, 20], we conduct a comprehensive phylogenetic study of *Goodyera* based on DNA sequence data of ITS and two plastid regions (*trnL-F*, *matK*) in this study, with the aim of assessing the monophyly of *Goodyera* and shed light on its infrageneric relationships.

## Materials and Methods

### Ethics statement

All the samples were collected and processed in their respective countries, for all samples from China, DNA extraction and sequencing was done from live or fresh silica gel dried specimens, for all samples from outside China, FTA cards were used to collect the plant extract for DNA extraction and sequencing. So none was taken out of its respective country, none of the species studied belongs to rare, endangered or threatened species according to IUCN, all orchids studied during the current study are not included under CITES Appendix II, none of the samples was collected within protected areas, hence no permission was needed.

### Taxon sampling

In total, we analysed 132 samples representing 35 species of *Goodyera* and 27 additional genera/48 species of related genera (55 accessions), including 64 sequences from GenBank. *Pterostylis longifolia* (Pterostylidinae) and *Chloraea gaudichaudii* (Chloraeinae) were chosen as outgroups on the basis of previous phylogenetic studies [23, 28]. Five species of *Goodyera* [*Goodyera brachystegia*, *G*. *fusca*, *G*. *makuensis* Ormerod, *G*. *malipoensis* Q. X. Guan & S. P. Chen and *G*. *wuana*] from China could not be sampled in spite of repeated attempts. Voucher specimens were deposited at the Herbarium of East China Normal University (HSNU) and the Herbarium of Taiwan Forestry Research Institute (TAIF). Detailed voucher information is provided in S1 Table.

### DNA extraction, amplification and sequencing

Genomic DNA was extracted from 10 mg of fresh or silica-dried tissue using a modified CTAB method [31]. Amplification was carried out on in a TAKARA TP600 thermocycler (TAKARA BIO INC, Japan) using 50 μl reactions containing 25 μl 2× Taq PCR Master Mix (BIOMIGA, China), 17.5 μl ddH_2_O, 2.5 μl of each primer (10 μM) and 2.5 μl of target DNA template (0.5 ng/μl). The ITS and *trnL-F* regions were amplified with two primers, but *matK* was amplified using 5 primers. The primers and amplification protocols for each DNA region are listed in S2 Table. PCR products were purified using a PCR purification kit (BIOMIGA, China).

For each region, both strands were sequenced with the same primers as for the amplification, except for *trnL-F* for which two internal primers were used. Sequencing for this work was outsourced to Invitrogen Biotechnology Corporation (Shanghai, China), Majorbio Bio-Pharm Technology Corporation Limited (Shanghai, China) and the Beijing Genomics Institute (BGI, China). All sequences have been submitted to GenBank and accession numbers are listed in S1 Table.

### Phylogenetic analyses

Sequences were firstly assembled and edited with Seqman (DNA STAR package, Madison, WI, USA) [32], aligned with Mega 5 [33] and then adjusted manually. Three datasets, namely ITS, the combined chloroplast dataset (*matK* and *trnL-F*) and the combined nuclear and chloroplast DNA sequences (ITS, *matK* and *trnL-F*) were analysed using Bayesian inference (BI), maximum parsimony (MP) and maximum likelihood (ML); all characters were treated as unordered and equally weighted. Indels were treated as missing data.

The maximum parsimony (MP) analyses were performed with PAUP* version 4.0b10 [34]. A heuristic search with 1000 random addition sequence replicates, tree bisection-reconnection (TBR) branch swapping and the MulTrees (saving multiple trees in memory) option were performed. Bootstrap values were generated with 1000 bootstrap replicates with TBR branch swapping, with each replicate performing 100 random-addition sequence replicates and a limit of 1000 trees. Homoplasy levels were assessed by means of the consistency index (CI) and the retention index (RI). For the ML analyses, MrModelTest 2.3 [35] was used to select the most suitable model under the Akaike information criterion (AIC) [36]. The GTR+I+G model was selected as the best-fit model by MrMTgui 1.0 [37] for all datasets. Then the models were added in a command block after the data in the NEXUS file. A total of 1000 bootstrap replicates were performed using Garli v0.951-GUI [38]. Other parameters were set as default for the Garli searches. PAUP * version 4.0b10 was used for exporting tree files. Both for the MP and ML analyses, bootstrap values over 89% were considered as high support, between 71% and 89% moderate support and below 71% as weak support. The Bayesian inference analyses were conducted with MrBayes 3.1.2 [39]. GTR+I+G was selected as the best-fit model as for the ML analyses for all datasets. The analyses consisted of 3,000,000 generations of four simultaneous Monte Carlo Markov chains. We increased the number of generations until the average deviation of split frequencies fell below 0.01 [40]. Trees were sampled every 1000 generations; the samples prior to stationary were discarded as burn-in using Tracer v. 1.5 and the remaining trees were used to build a majority-rule consensus tree on which the posterior probabilities (PP) were shown. We defined PP values above 0.90 as high support, between 0.80 and 0.89 as moderate support, and below 0.79 as weak support.

### Homogeneity test

Homogeneity between the ITS data and the combined chloroplast datasets (*trnL-F* and *matK*) was tested following Farris et al. [41] using the incongruence length difference (ILD) test, as implemented in PAUP* version 4.0b10 [34].

## Results

### Characteristics of sequence data and inferred phylogenetic trees

One hundred and forty-three ITS, 90 *trnL-F* and 82 *matK* sequences were newly generated in this study. All these sequences have been submitted to GenBank. Sixty-four sequences were added to our analysis from GenBank. In total, we analysed 379 sequences of 187 accessions (132 of *Goodyera* and 55 of other genera).

**Fig 1:**
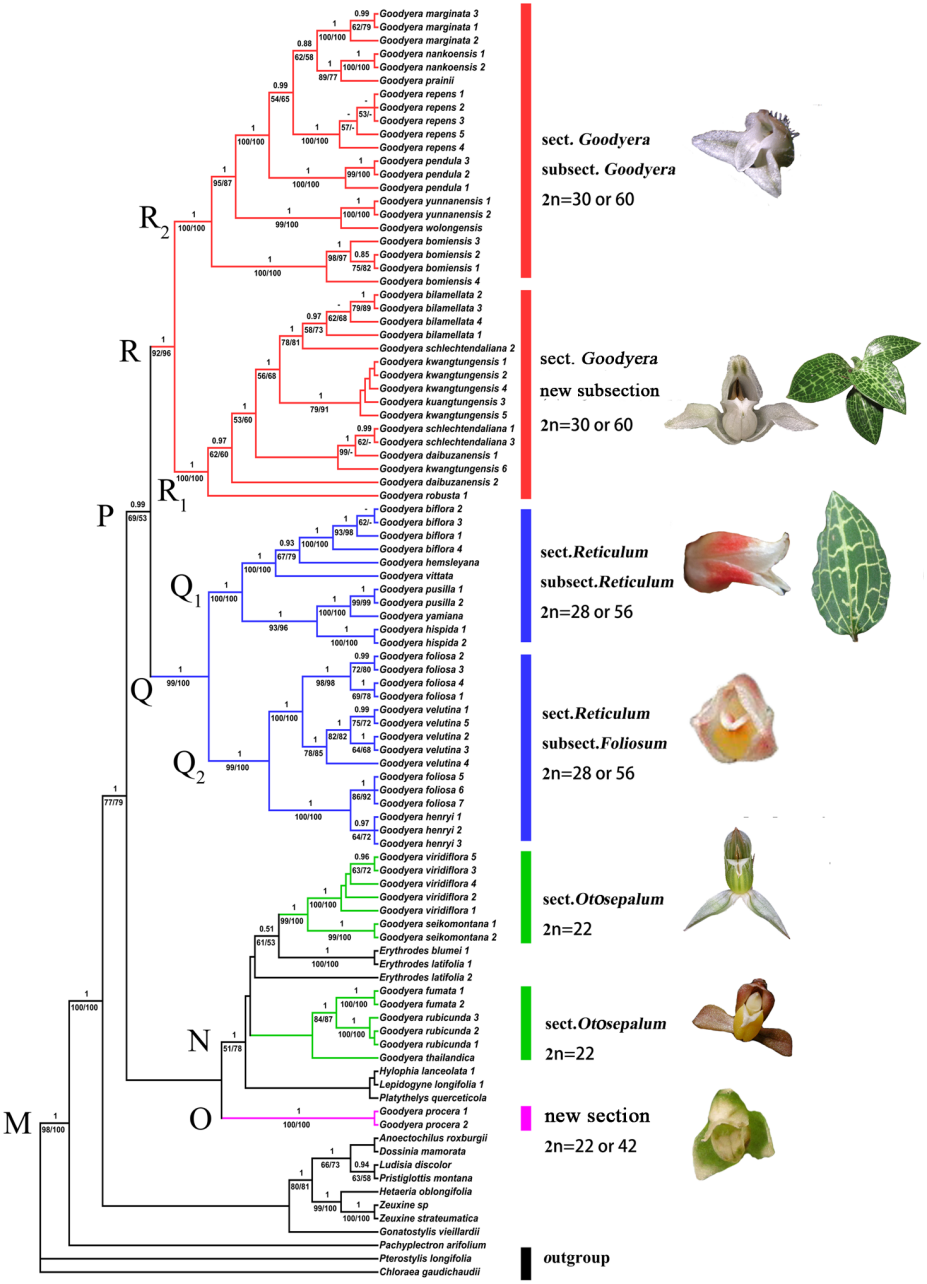
Strict consensus tree from the MP analysis based on ITS, *trnL-F* and *matK* data. PP≥0.5 are shown above the branches and bootstrap values ≥50% are shown below the branches (MP/ML; dashes mean no support). Groups are labelled to the right.

Details of three datasets are shown in Table 2. For all datasets, the three phylogenetic methods yielded similar phylogenetic patterns, but the MP trees were the most resolved. Posterior Probabilities from the BI analysis and bootstrap values from both the MP and ML analysis are shown on the MP trees (Fig 1, S1 and S2 Figs).

**Table 2:** Characteristics of individual and combined datasets.

Dataset	No. of taxa	Aligned length (bp)	Variable sites	Parsimony-informative sites	Number of most-parsimonious trees	MP tree length	CI	RI	GARLI ML score
ITS	185	818	162	347	141	1662	0.5078	0.4922	-10241.7098
			(19.80%)	(42.42%)					
*trnL-F* & *matK*	97	3717	577	588	65	2236	0.6561	0.3439	-12041.6911
			(15.81%)	(15.82%)					
ITS, *trnL-F* & *matK*	95	4507	725	819	12	3180	0.6314	0.3686	-24047.4913
			(16.08%)	(18.17%)					

### Nuclear DNA dataset analysis

The ITS dataset included 183 ingroup taxa, two outgroups and 818 characters, of which 162 (19.80%) were variable and 347 (42.42%) were parsimony-informative. The analysis found 141 equally shortest trees with a length of 1662 steps, CI = 0.5078 and RI = 0.4922. The strict consensus of the 141 trees is shown in S1 Fig. The genus *Goodyera* is split into several clades. *Erythrodes* Bl., *Platythelys* Garay, *Microchilus* C. Presl, *Kreodanthus* Garay, *Lepidogyne* Bl. and *Hylophila* Lindl. are nested with species of *Goodyera* in clade B. Clade C consists of two sister subclades: C_1_ and C_2_, while *Goodyera vittata* is embedded in clade C_1_. The complex of *G*. *schlechtendaliana*, containing five morphologically confusing taxa (*G*. *schlechtendaliana*, *G*. *robusta* Hook. f., *G*. *daibuzanensis*, *G*. *kwangtungensis*, *G*. *bilamellata*) formed a clade with high support value (PP = 1, MP = 99%, ML = 99%). *Goodyera repens* and other 10 species from high elevations (*G*. *rosulacea* Y. N. Lee and *G*. *tesselata* G. Lodd are not recorded in China) formed a moderate to highly supported clade E (PP = 1, MP = 91%, ML = 86%). Three foreign species [*G*. *pubescens*, *G*. *oblongifolia*, *G*. *brachyceras* (A. Rich. & Galeotti) Garay & G. A. Romero] clustered with clade E, but lacking support.

### Combined chloroplast DNA dataset analysis

The tree inferred by using two plastid markers (*trnL-F* & *matK*) was better resolved than the ITS tree. The plastid dataset included 95 ingroup taxa and two outgroups and consisted of 3717 characters of which 577 (15.81%) were variable and 588 (15.82%) were parsimony-informative. The analysis found 65 most parsimonious trees of 2236 steps, CI = 0.6561 and RI = 0.3439. The strict consensus tree from the MP analysis is shown in S2 Fig. *Erythrodes*, *Platythelys*, *Ludisia* A. Rich., *Lepidogyne* and *Hylophila* formed clade H with *Goodyera* sect. *Otosepalum* except *G*. *procera*, gaining different levels of support from three analyses (PP = 1, MP = 55%, ML = 76%). *Goodyera procera* formed an independent clade (I) with high support. The rest of the species formed a clade (J) (PP = 0.99, MP = 76%, ML = 72%) in turn split into two well supported clades: clade K and clade L. Clade K (PP = 1, MP = 98%, ML = 99%) with species of sect. *Reticulum* split into two subclades K_1_ and K_2_, clade L (PP = 1, MP = 88%, ML = 89%) with the species of sect. *Goodyera* split into subclades L_1_ and L_2_.

### Combined nuclear and chloroplast DNA dataset analysis

The result of the ILD test for the nrDNA and combined cpDNA showed incongruence between the two datasets (P = 0.01). However, the support of branches increased in the combined tree and the incongruence might disappear with more data [41]. So we included the combined dataset in our analysis. The combined dataset of the three markers (ITS, *trnL-F* & *matK*) had 4033 aligned characters, of which 725 (16.08%) were variable and 819 (18.17%) were parsimony-informative. The analysis found 12 most parsimonious trees with a length of 3180 steps, CI = 0.6314 and RI = 0.3686. The strict consensus of the 12 trees from the combined MP analysis is shown in Fig 1. It showed a similar topology to the plastid DNA tree with the support values of some main clades increased, and it was more resolved than the ITS analysis. The combined tree of three markers indicated that *Goodyera* is closely allied to *Erythrodes*, *Platythelys*, *Lepidogyne* and *Hylophila* and these genera were more close to sect. *Otosepalum*. *Goodyera procera* formed an independent clade (O) (PP = 1, MP = 100%, ML = 100%). The strongly supported clade Q (PP = 1, MP = 99%, ML = 100%) and clade R (PP = 1, MP = 92%, ML = 96%) each consisted of two subclades (subclades Q_1_, Q_2_ and subclades R_1_, R_2_, respectively). *Goodyera vittata* and *G*. *biflora* nested in subclade Q_1_ with species of sect *Goodyera*, subsect. *Reticulum*. Three species viz., *G*. *foliosa*, *G*. *velutina* and *G*. *henryi* Rolfe formed a well supported subclade Q_2_ (PP = 1, MP = 99%, ML = 100%). The complexes of *G*. *schlechtendaliana* (*G*. *schlechtendaliana*, *G*. *daibuzanensis*., *G*. *bilamellata G*. *kwangtungensis* and *G*. *robusta*) and *G*. *repens* (*G*. *nankoensis*, *G*. *prainii* Hook. f., *G*. *marginata* Lindl., *G*. *pendula* Maxim., *G*. *yunnanensis* Schltr., *G*. *wolongensis* and *G*. *bomiensis*) each formed a strongly supported subclade (R_1_ and R_2_, respectively).

## Discussion

### Circumscription of *Goodyera*

Goodyerinae had been recognized as a well-defined group by several authors [21, 28] using molecular data from both chloroplast and nuclear markers. However, within the subtribe especially in *Goodyera*, the evolutionary relationships between taxa remain unresolved. Pridgeon et al. [3] pointed out that circumscription of *Goodyera* was problematic and that a better understanding of the infrageneric phylogeny was needed.

In this study, *Goodyera* turns out to be polyphyletic, in contrast with previous studies [17, 18], which included only a small number of samples from other genera of Goodyerinae. Our analysis has revealed that there are at least six genera viz., *Erythrodes*, *Kreodanthus*, *Microchilus*, *Platythelys*, *Lepidogyne* and *Hylophila* that have a close relationship with *Goodyera*. In all our trees, these genera are embedded within the *Goodyera* clade. *Erythrodes*, *Lepidogyne* and *Hylophila* do share some morphological characters (such as lack of marked leaf surface) with *Goodyera* subsect. *Otosepalum*. *Erythrodes*, *Lepidogyne*, *Hylophila* and subsect. *Otosepalum* are all distributed in Malaysia, Indonesia, Philippines and some other tropical Asia areas. However, *Erythrodes* differs in the flowers, which always have a spurred lip lacking the numerous setose appendages often present in different species of *Goodyera*. *Hylophila* is distinguishable from *Goodyera* by its flowers with a large scrotiform lip, whereas *Lepidogyne* differs from *Goodyera* by its short, thick stems, and a projecting plate under the stigma of flowers. *Platythelys* differs in having smaller, fleshy flowers with a broad, flat, elliptic to suborbicular rostellum and the species of this genus are restricted to the tropics and subtropics of the New World. Further research is needed to clarify the relationships of *Goodyera* with other genera in Goodyerinae.

Our results are identical with previous researches, such as Juswara [20]. In her phylogenetic tree inferred from ITS, *trnL-F* and *rpl16*, *Goodyera* turned out to be polyphyletic. But it included only small number of species and the placement of *G*. *vittata* is totally different from us, which could be a case of misidentifications in her research. and because of sample limitation. So, further studies with more samples especially other genus of Goodyerinae are still needed.

### Infrageneric relationships of *Goodyera*

According to morphological characters, a small number of *Goodyera* species were assigned to two newly established sections [10]. Chung [18] divided the *Goodyera* species in Taiwan into three sections and seven subsections based on ITS and chromosome numbers (Table 1). The relationships revealed by three DNA loci in this study (Fig 1, S1 and S2 Figs) do not agree with the infrageneric classification of previous studies [10, 18].

The species *Goodyera procera* formed a clade (Clade O in Fig 1) with high support. *G*. *procera* has narrowly ovate-elliptic leaves, spike inflorescence with very dense flowers that are not usually secund and mainly grows beside streams in forests instead of in humus like most other *Goodyera* species. In all our molecular analyses, *G*. *procera* did not form a clade with other species of sect. *Otosepalum* and sometimes has different chromosome numbers (2*n* = 22 or 42) with respect to other species of sect. *Otosepalum* (2*n* = 22 or 44). It should be treated as a separate section by taking all the molecular and morphological differences into consideration (H.Z. Tian & C. Hu, unpublished manuscript).

Five species are included in clade N (*Goodyera thailandica* Seidenf., *G*. *fumata*, *G*. *rubicunda* (Blume) Lindl., *G*. *seikoomontana* Yamam. and *G*. *viridiflora*). As we get from the phylogenetic trees, these species of *Goodyera* have a very close relationship with some other genera (*Erythrodes*, *Lepidogyne*, *Hylophila* and *Platythelys*). These species vary considerably in their flowers and vegetative parts. *Goodyera seikoomontana* and *G*. *viridiflora* share many similarities (big flowers, reflexed lateral sepals and long pollinia) and differ a lot from the other three species. The flowers of *G*. *fumata*, *G*. *rubicunda* and *G*. *thailandica* do share a similar size and shape of leaves and inflorescence with *Erythrodes* but differ in their absence of a spur. In this study, we assign these five species to sect. *Otosepalum* of *Goodyera* because of their saccate labellum. Further studies such as morphology and molecular phylogeny including more species within *Goodyera* and related genera need to be conducted to clarify their relationships.

In Fig 1, most species of clade Q have silver or gold veins and the lateral sepals are not opened. Clade Q is related to sect. *Reticulum* S.W. Chung & C.H. Ou. It is split into two well supported clades (Q_1_ and Q_2_). Most species of clade Q_1_ have reticulate venation on leaves, lateral sepals are not opened and lip sacs do not extend up to the lateral sepals. Morphologically, these species show marked differentiating characters and can be easily distinguished from each other as well as other species of *Goodyera*. Chung [18] established two subsections (subsect. *Biflora* and subsect. *Reticulum*) based on ITS for these species with a single species *G*. *biflora* forming subsect. *Biflora*. This is not supported by any of our ITS, plasted or combined analyses (S1 Fig). *Goodyera biflora* was included in subsect. *Biflora* by having long tubular flowers and reticulate venation on the leaves [18]. In fact, between the long tubular flowers of *G*. *biflora* and the short tubular flowers of *G*. *hispida*, there are some transitional species such as *G*. *vittata* and *G*. *hemsleyana*. So this well supported clade Q_1_ can be merged into one subsection (subsect. *Reticulum*).

Clade Q_2_ is related to subsect. *Foliosum*. Some problems still remained in this clade. *Goodyera foliosa* formed two strongly supported clades with *G*. *velutina* and *G*. *henryi* respectively. Though *G*. *velutina* can be easily distinguished from *G*. *foliosa* by its white or pink mid-vein on leaf, the phylogenetic trees show complex relationships among them, which requires further studies.

All species in clade R can be placed in sect. *Goodyera* by morphological data and chromosome number (2*n* = 30 or 60). Only in this clade are there some species epiphytic, such as *G*. *bilamellata*, *G*. *pendula*, *G*. *pranii*, and sometimes *G*. *schlechtendaliana*.

Clade R split into two clades, R_1_ and R_2_. Clade R_1_ contained five species known as a species complex that share many similarities: white flowers like a flying dove and white marking on the upper surface of leaves (*G*. *billamelata* and *G*. *robusta* are exceptions). Due to the lack of abrupt interspecific variations, it is difficult to distinguish the species of this complex. In the previous studies [4, 5], some species were merged. But because of lacking molecular data, those treatments are debatable. In this study, the phylogenetic trees show that this complex is not well resolved, and more samples and studies are needed. But it can be confirmed is that these species form a well-supported clade and should be separated from subsect. *Goodyera* by establishing a new subsection (H. Z. Tian & C. Hu, unpublished manuscript).

All species in clade R_2_ share two features: almost glabrous inside the labellum and distribution at higher elevations. It is reasonable to treat R_2_ as a unique subsection merged with species of subsect. *Recurvum*. The name of this subsection should be *Goodyera* because the type species of the genus *Goodyera*, *G*. *repens*, forms part of this clade.

### Biogeography of *Goodyera*

The pollinarium fossil of *Meliorchis caribea* from Dominican Republic indicated that a minimum age of 15–20 Myr can be assigned to the subtribe Goodyerinae [42].

**Fig 2:**
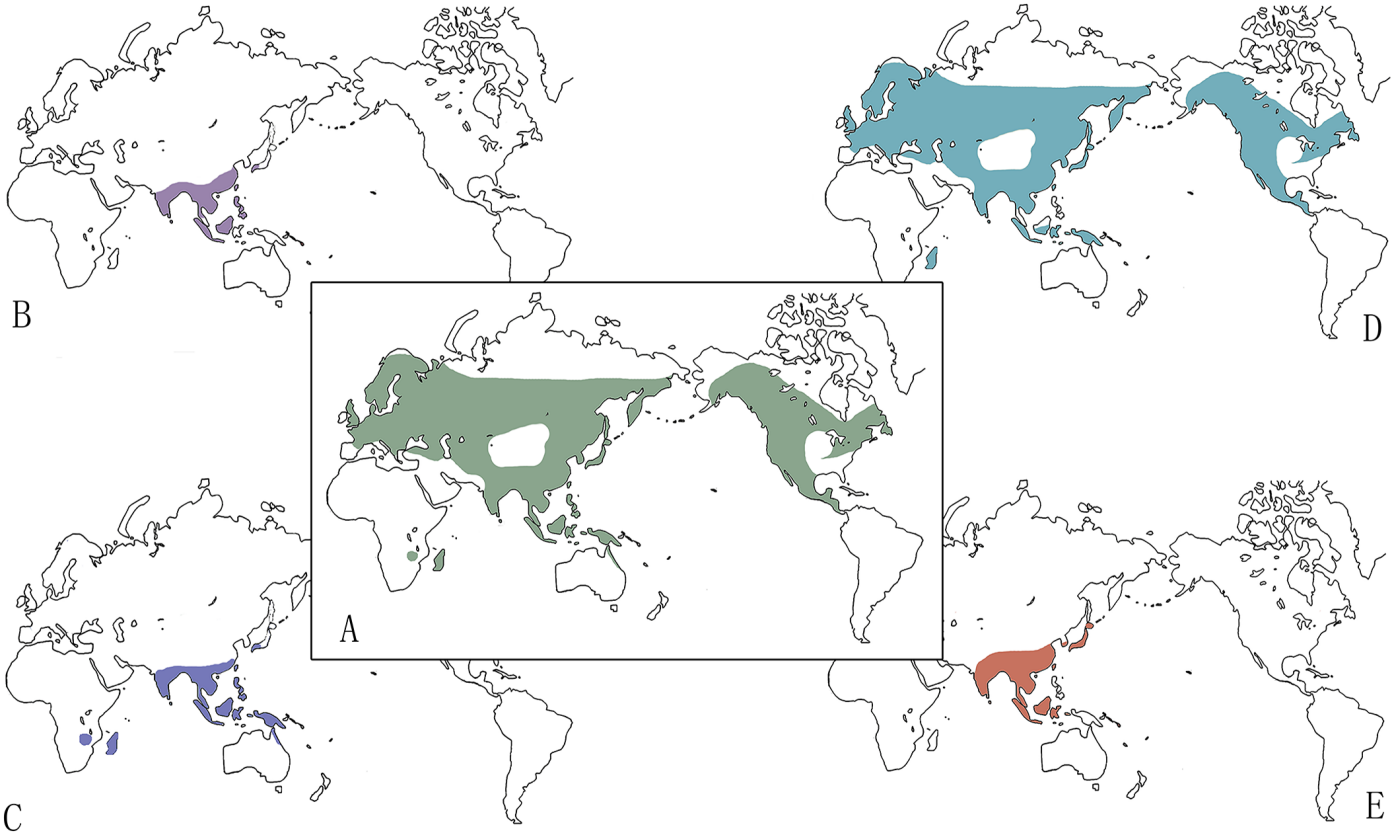
The distribution of *Goodyera*. A. the genus *Goodyera*; B. The new section (H.Z. Tian & C. Hu, unpublished manuscript); C. Sect. *Otosepalum*; D. Sect. *Goodyera*; E. Sect. *Reticulum*.

The genus *Goodyera* is worldwide and species are mostly distributed in temperate and tropical regions of Asia. Tian [15] divided the distribution patterns of *Goodyera* species from China into six types (North Temperate, Tropical Asia, Tropical Asia to Tropical Australasia, Tropical Asia to East Asia, East Asia and Endemic to China). As we can see from Fig 2, the new section of *G*. *procera* shows Tropical Asia distribution (Fig 2B). Sect. *Otosepalum* is mainly distributed in Tropical Asia and some species such as *G*. *rubicunda*, *G*. *fumata* southwards to Tropical Australia and Pacific Islands (Fig 2C). Meanwhile, due to the tropical habitat of these species, they are always tall and robust. Sect. *Reticulum* ranges from Tropical Asia to East Aisa (Fig 2E). The distribution of sect. *Goodyera* reflects almost all part of the genus (Fig 2D) and this section comprises many alpine species as well as epiphytic species. *Goodyera repens* is the most widely distributed species in *Goodyera*. Tsiftsis and Papaioannou [43] pointed out that the southern distribution limits of *G*. *repens* is indirectly affected by soil variables through the establishment of mycorrhizal symbiosis and its distribution is found to be negatively correlated with the nutrient content of the soil. It is worthy to be mentioned that the microhabitat is really important for species differentiation in *Goodyera*. Most Chinese endemic species in subsect. *Goodyera* are affinis with *G*. *repens* and mainly distributed in Hengduan Mountain with relatively high altitude.

To know the geographic tracks of *Goodyera* species, more samples of Goodyerinae especially species from other parts of the world are needed. More comprehensive biogeographic analysis such as divergence time and ancestral area reconstructions can reveal details about this genus.

## Conclusions

According to this study, *Goodyera* is polyphyletic and can be divided into four sections: sect. *Otosepalum* characterized by leaves without markings and lateral sepals reflexed; the single species *G*. *procera* forms a new section and characterized by green leaves and nearly spicate inflorescence; sect. *Reticulum* with subsect. *Reticulum* (having golden venation on the leaves) and subsect. *Foliosum* containing *G*. *foliosa*, *G*. *henryi* (three pale veins on the leaves) and *G*. *velutina* (one white, golden or pink mid-vein); sect. *Goodyera* with subsect. *Goodyera* (smaller flowers with almost glabrous lip sac) and a new subsection (white dove-like flowers with papillose lip sac). Further studies are needed especially in some species complexes.

## Supporting Information

S1 Fig The strict consensus tree from the MP analysis based on ITS data. Posterior probalities ≥0.5 (from the Bayesian analysis) are shown above the branches and bootstrap values ≥50% are shown below the branches (MP/ML; dashes mean no support). Groups are labelled to the right.
(TIF)

S2 Fig The strict consensus tree from the MP analysis based on *trnL-F* and *matK* data. Posterior probabilities ≥0.5 (from the Bayesian analysis) are shown above the branches and bootstrap values≥50% are shown below the branches (MP/ML; dashes mean no support). Groups are labelled to the right.
(TIF)

S1 Table Details of material included in this study. (DOCX)

S2 Table Primers used for amplification and sequencing. (DOCX)
