# Supplementary figures and images for: Phylogenetic Analysis of a ‘Jewel Orchid’ Genus Goodyera (Orchidaceae) Based on DNA Sequence Data from Nuclear and Plastid Regions
Source: PLoS One. 2016 Feb 29;11(2):e0150366. doi: 10.1371/journal.pone.0150366 (PMC4771202; doi:10.1371/journal.pone.0150366)

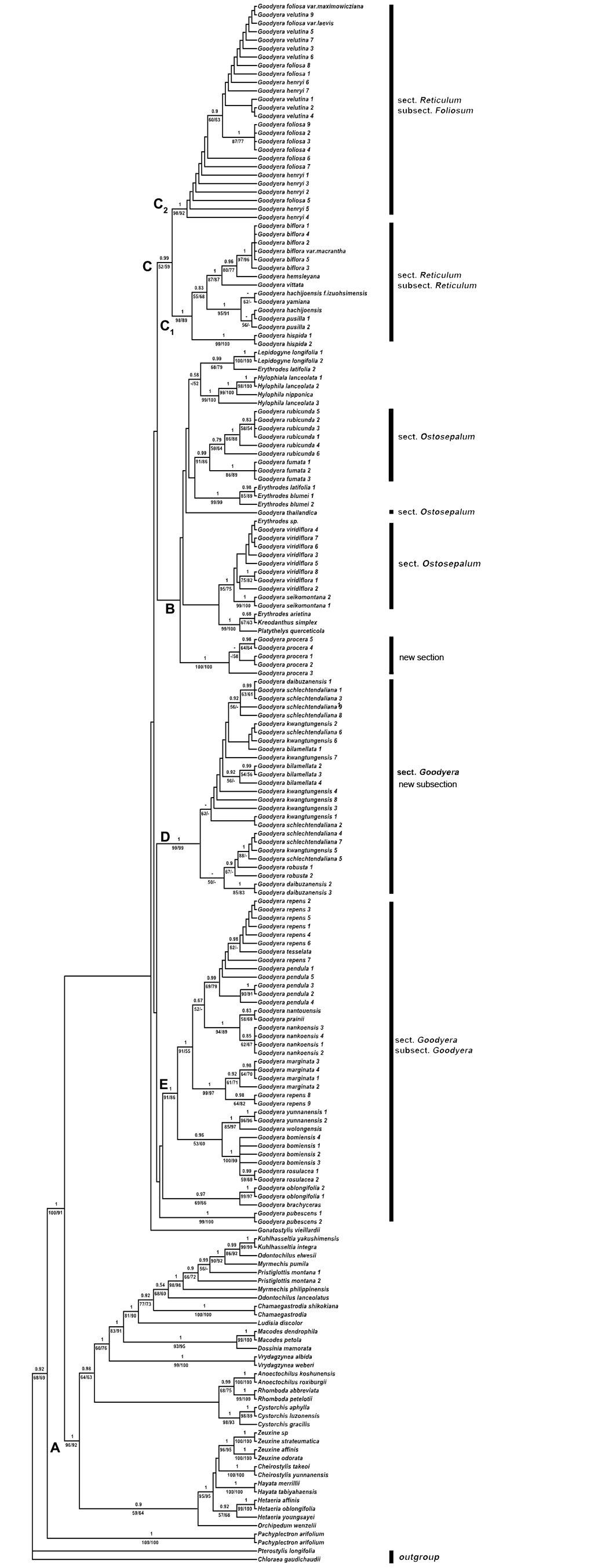

Supplement: S1 Fig — Posterior probalities ≥0.5 (from the Bayesian analysis) are shown above the branches and bootstrap values ≥50% are shown below the branches (MP/ML; dashes mean no support). Groups are labelled to the right. (TIF) [file pone.0150366.s001.tif]

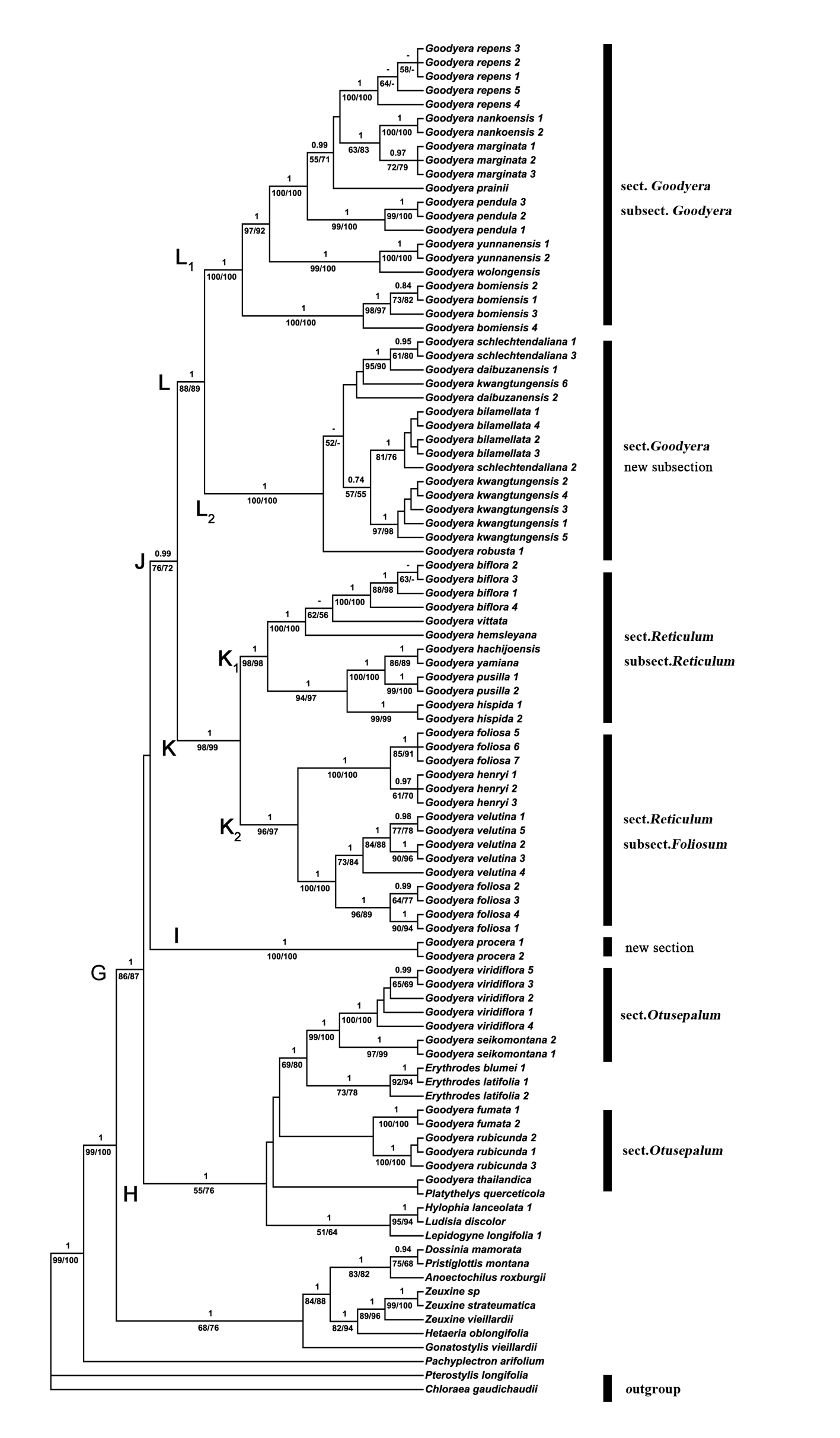

Supplement: S2 Fig — Posterior probabilities ≥0.5 (from the Bayesian analysis) are shown above the branches and bootstrap values≥50% are shown below the branches (MP/ML; dashes mean no support). Groups are labelled to the right. (TIF) [file pone.0150366.s002.tif]
