# Supplementary material for: Phylogenetic Analysis of a ‘Jewel Orchid’ Genus Goodyera (Orchidaceae) Based on DNA Sequence Data from Nuclear and Plastid Regions
Source: PLoS One. 2016 Feb 29;11(2):e0150366. doi: 10.1371/journal.pone.0150366 (PMC4771202; doi:10.1371/journal.pone.0150366)
Supplement: S1 Table — (DOCX) [file pone.0150366.s003.docx]

**Table S1 Details of materials included in this study**

| **Taxon** | **Voucher/Collector/locality** | **GeneBank Accession** | | |
| --- | --- | --- | --- | --- |
|  |  | **ITS** | ***trn*L-F** | ***mat*K** |
| *Anoectochilus roxburghii* (Wallich) Lindley*** | b02/Hu/Liuxihe, Guangdong, China | EU817408 | KT385493 | EU817409 |
| *Anoectochilus* *koshunensis* Hayata | 357/Chung/Mt. Qingshui, Hualian County, Taiwan, China | KT334331 | - | - |
| *Chamaegastrodia* sp. | 457/Chung/Langyaxueshan, Tengchong, Yunnan, China | KT334332 | - | - |
| *Chamaegastrodia shikokiana* Makino & F. Maekawa | - | JN166061 | - | - |
| *Cheirostylis takeoi* (Hayata) Schlechter | 334/Chung/Taigang, Xinzhu County, Taiwan, China | KT343977 | - | - |
| *Cheirostylis yunnanensis* Rolfe | 274/Chung/Daxueshan, Lincang, Yunnan, China | KT343978 | - |  |
| *Chloraea gaudichaudii* Brongn. *** | - | FR832116 | AJ409383 | AJ310004 |
| *Cystorchis luzonensis* Ames | 438/Chung/Baler, Philippines | KT343979 | - | - |
| *Cystorchis aphylla* Ridl. | - | JN166062 | - | - |
| *Cystorchis gracilis* (Hook. f. ) Holttum | - | JN166063 | - | - |
| *Dossinia marmorata* C. Morren*** | 277/Chung/Malaysia | KT343980 | KT385494 | AJ543947 |
| *Erythrodes blumei* (Lindl. ) Schltr. 1 | b109/Tian/Wulai, Taibei, Taiwan, China | KT343982 | KT385495 | KT385583 |
| *E. blumei* 2 | 440/Chung/Shouka, Taitong County, Taiwan, China | KT343981 | - | - |
| *Erythrodes latifolia* Blume1*** | 441/Chung/Yunnan, China | KT343983 | KT385496 | JN166025 |
| *E. latifolia* 2 | 553/Chung/Halimun, Indonesia | KT343984 | KT385497 | KT385584 |
| *Erythrodes* sp. | 49/Chung/Cibodas, Indonesia | KT343985 | - | - |
| *Gonatostylis vieillardii* (Rchb. f. ) Schltr*** | - | FJ473325 | AJ409416 | AJ310034 |
| *Goodyera biflora* (Lindl. ) Hook. f. 1 | 359/Chung/Xiakeluo, Xingzhu County, Taiwan, China | KT343986 | KT385501 | KT385588 |
| *G. biflora* 2 | b03/Hu/Tianmu Mountainshan, Lingan, Zhejiang, China | KT343988 | KT385499 | KT385586 |
| *G. biflora* 3 | b41/Hu & Li/Tianpingshan Mountain, Sangzhi, Hunan, China | KT343989 | KT385500 | KT385587 |
| *G. biflora* 4 | a12/Tian/Cibagou, Xizang, China | KT343987 | KT385498 | KT385585 |
| *G. biflora* 5 | **-** | AF366894 | **-** | **-** |
| *G. biflora* var. *macrantha* | **-** | HM021585 | **-** | **-** |
| *Goodyera bilamellata* Hayata 1 | 183/Chung/Mt. Qhingshui, Hualian, Taiwan, China | KT343990 | KT385502 | KT385589 |
| *G. bilamellata* 2 | 298/Chung/Mt. Zhulushan, Taibei, Taiwan, China | KT343991 | KT385503 | KT385590 |
| *G. bilamellata* 3 | 630/Chung/Meifeng, Nantou, Taiwan, China | KT343993 | KT385505 | KT385592 |
| *G. bilamellata* 4 | 628/Chung/Simakusi, Xingzhu County, Taiwan, China | KT343992 | KT385504 | KT385591 |
| *Goodyera bomiensis* K. Y. Lang 1 | a15/Tian/Pailong, Linzhi county, Xizang, China | KT343995 | KT385507 | KT385594 |
| *G. bomiensis* 2 | b129/Hu & Li/Huoshan County, Anhui, China | KT343996 | KT385508 | KT385595 |
| *G. bomiensis* 3 | c14/Tang/Luojishan, Xichang, Sichuan, China | KT343997 | KT385509 | KT385596 |
| *G. bomiensis* 4 | 588/Chung/Xiakelo, Xinzhu County, Taiwan, China | KT343994 | KT385506 | KT385593 |
| *G. brachyceras* (A. Rich. & Galeotti) Garay & G. A. Romero | **-** | AM778169 | **-** | **-** |
| *G. daibuzanensis* Yamam. 1 | a11/Tian/Cibagou, Chayu, Xizang, China | KT343999 | KT385510 | KT385597 |
| *G. daibuzanensis* 2 | b98/Tian/Jingshuiying, Pingdong County, Taiwan, China | KT344000 | KT385511 | KT385598 |
| *G. daibuzanensis* 3 | 527/Chung/Mt. southern Chatian, Taibei, Taiwan, China | KT343998 | **-** | **-** |
| *Goodyera foliosa* (Lindl. ) Benth. Ex C. B. Clarke 1 | b96/Tian/Wulai, Taibei, Taiwan, China | KT344009 | KT385518 | KT385605 |
| *G. foliosa* 2 | b11/Hu/Yezhutang, Malipo, Yunnan, China | KT344003 | KT385512 | KT385599 |
| *G. foliosa* 3 | b12/Hu/Yezhutang, Malipo, Yunnan, China | KT344004 | KT385513 | KT385600 |
| *G. foliosa* 4 | b47/Hu/Maoershan, Guiling, Guangxi, China | KT344005 | KT385514 | KT385601 |
| *G. foliosa* 5 | b78/Ye/Caiyanghe, Simao, Yunnan, China | KT344006 | KT385515 | KT385602 |
| *G. foliosa* 6 | b80/Ye/Caiyanghe, Simao, Yunnan, China | KT344007 | KT385516 | KT385603 |
| *G. foliosa* 7 | b81/Ye/Simao, Yunnan, China | KT344008 | KT385517 | KT385604 |
| *G. foliosa* 8 | 401/Chung/Tam Dao, Viet Nam | KT344001 | **-** | **-** |
| *G. foliosa* 9 | 81/Chung/Lanyu Island, Taidong County, Taiwan, China | KT344002 | **-** | **-** |
| *G. foliosa* 10 | **-** | HM140998 | **-** | **-** |
| *G. foliosa* 11 | - | HM140989 | **-** | **-** |
| *Goodyera fumata* Thwaites 1 | 443/Chung/Hapen, Taibei, Taiwan, China | KT344010 | KT385519 | KT385606 |
| *G. fumata* 2 | b125/Tian & Dong/Wuzhishan, Hainan, China | KT344011 | KT385520 | KT385607 |
| *G. fumata* 3 | b23/Hu/Xishuangbanna Botanical Garden, Yunnan, China | KT344012 | **-** | **-** |
| *Goodyera hachijoensis* Yatabe 1 | 199/Chung/Mt. Qingshui, Hualian County, Taiwan, China | KT344013 | **-** | **-** |
| *G. hachijoensis* 2 | 46/Chung/Cibodas, Indonesia | **-** | KT385521 | KT385608 |
| *G. hachijoensis* f. *izuohsimensis* | - | HM140990 | **-** | **-** |
| *Goodyera hemsleyana* King & Pantl. | b14/Hu/Dayakou, Malipo, Yunnan, China | KT344014 | KT385522 | KT385609 |
| *Goodyera henryi* Rolfe 1 | a10/Tian/Cibagou, Xizang, China | KT344017 | KT385523 | KT385610 |
| *G. henryi* 2 | b32/Hu/Sanjiang Ecological Tourist Area, Wenchuan, Sichuan, China | KT344018 | KT385524 | KT385611 |
| *G. henryi* 3 | b36/Hu & Li/Emeishan, Sichuan, China | KT344019 | KT385525 | KT385612 |
| *G. henryi* 4 | 554/Chung/Cibodas, Indonesia | KT344016 | **-** | **-** |
| *G. henryi* 5 | 393/Chung/Mt. Fansipan, Viet Nam | KT344015 | **-** | **-** |
| *G. henryi* 6 | **-** | HM021586 | **-** | **-** |
| *G. henryi* 7 | **-** | AF366895 | **-** | **-** |
| *Goodyera hispida* Lindl. 1 | b86/Tian/Motuo, Xizang, China | KT344020 | KT385526 | KT385613 |
| *G. hispida* 2 | c12/Jiang | KT344021 | KT385527 | KT385614 |
| *Goodyera kwangtungensis* C. L. Tso 1 | a18/Tian/Nanling, Guangdong, China | KT344024 | KT385528 | KT385615 |
| *G. kwangtungensis* 2 | b30/Hu & Li/Jinfo Mountain, Nanchuan, Chongqing, China | KT344027 | KT385531 | KT385618 |
| *G. kwangtungensis* 3 | b37/Hu/Emeishan, Sichuan, China | KT344028 | KT385532 | KT385619 |
| *G. kwangtungensis* 4 | b44/Hu/Maoershan, Guangxi, China | KT344029 | KT385533 | KT385620 |
| *G. kwangtungensis* 5 | b16/Hu/Dayakou, Malipo, Yunan, China | KT344025 | KT385529 | KT385616 |
| *G. kwangtungensis* 6 | b17/Hu/Dayakou, Malipo, Yunnan, China | KT344026 | KT385530 | KT385617 |
| *G. kwangtungensis* 7 | 184/Chung/Mt. Qhingshui, Hualian, Taiwan, China | KT344022 | **-** | **-** |
| *G. kwangtungensis* 8 | 276/Chung/Guangdong, China | KT344023 | **-** | **-** |
| *Goodyera marginata* Lindl. 1 | b06/Hu/Cangshan, Dali, Yunnan, China | KT344033 | KT385536 | KT385623 |
| *G. marginata* 2 | a13-4/Tian/Gangxiang, Bomi, Xizang, China | KT344032 | KT385535 | KT385622 |
| *G. marginata* 3 | 503/Chung/Chengdu, Sichuan, China | KT344030 | KT385534 | KT385621 |
| *G. marginata* 4 | 504/Chung/Emeishan, Sichuan, China | KT344031 | **-** | **-** |
| *Goodyera nankoensis* Fukuy. 1 | b33/Hu & Li/Emeishan, Sichuan, China | KT344036 | KT385537 | KT385624 |
| *G. nankoensis* 2 | b57/Ge/Gangxiang, Bomi, Xizang, China | KT344037 | KT385538 | KT385625 |
| *G. nankoensis* 3 | 340/Chung/Mt. Qingshui, Hualian County, Taiwan, China | KT344034 | **-** | **-** |
| *G. nankoensis* 4 | 498/Chung/Chengdu, Sichuan, China | KT344035 | **-** | **-** |
| *Goodyera nantoensis* Hayata | z-2/Chung/Mt. Lala, Taoyuan, Taiwan, China | KT344038 | **-** | **-** |
| *Goodyera oblongifolia* Raf. 1 | **-** | HM141005 | **-** | **-** |
| *G. oblongifolia* 2 | **-** | HM141004 | **-** | **-** |
| *Goodyera pendula* Maxim. 1 | a16/Tian/Nanling, Guangdong, China | KT344041 | KT385541 | KT385628 |
| *G. pendula* 2 | 629/Chung/Liyuan, Taitong County, Taiwan, China | KT344040 | KT385540 | KT385627 |
| *G. pendula* 3 | 525/Chung/Siyuanwukou, Yilan County, Taiwan, China | KT344039 | KT385539 | KT385626 |
| *G. pendula* 4 | **-** | HM141003 | **-** | **-** |
| *G. pendula* 5 | **-** | HM141002 | **-** | **-** |
| *Goodyera procera* (Ker Gawl. ) 1 | b01/Hu/Southern China Botanical Garden, Guangdong, China | KT344044 | KT385543 | KT385630 |
| *G. procera* 2 | b69/Tian & Dong/Wuzhishan, Hainan, China | KT344045 | KT385544 | KT385631 |
| *G. procera* 3 | 540/Chung | KT344043 | **-** | **-** |
| *G. procera* 4 | **-** | JN114515 | **-** | **-** |
| *G. procera* 5 | **-** | HM222488 | **-** | **-** |
| *Goodyera prainii* Hook. f. | 707-2/Tian/Tongbiguan, Yingjiang, Yunnan, China | KT344042 | KT385542 | KT385629 |
| *Goodyera pubescens* (Willd. ) R. Br. 1 | **-** | FJ473326 | **-** | **-** |
| *G. pubescens* 2 | **-** | AJ539519 | **-** | **-** |
| *Goodyera repens* (L. )R. Br. 1 | b48/Wei/Mt. Tianshan, Xinjiang, China | KT344050 | KT385549 | KT385636 |
| *G. repens* 2 | b49/Wei/Mt. Tianshan, Xinjiang, China | KT344051 | KT385550 | KT385637 |
| *G. repens* 3 | b50/Wei/Mt. Tianshan, Xinjiang, China | KT344052 | KT385551 | KT385638 |
| *G. repens* 4 | 632/Chung/Deqing County, Xizang, China | KT344048 | KT385547 | KT385634 |
| *G. repens* 5 | a13-1/Tian/Gangxiang, Bomi, Xizang, China | KT344049 | KT385548 | KT385635 |
| *G. repens* 6 | **-** | HM021555 | **-** | **-** |
| *G. repens* 7 | **-** | FJ473327 | **-** | **-** |
| *G. repens* 8 | **-** | JN114523 | **-** | **-** |
| *G. repens* 9 | **-** | JN114519 | **-** | **-** |
| *Goodyera robusta* Hook. f. 1 | 541/Chung/Mt. Fansipan, Sapa, Viet Nam | KT344054 | KT385552 | KT385639 |
| *G. robusta* 2 | 386/Chung/Mt. Fansipan, Sapa, Viet Nam | KT344053 | **-** | **-** |
| *Goodyera rosulacea* Y. N. Lee 1 | **-** | HM021558 | **-** | **-** |
| *G. rosulacea* 2 | **-** | HM021559 | **-** | **-** |
| *Goodyera rubicunda* (Blume) Lindl. 1 | b88/Tian/Bawangling, China | KT344060 | KT385555 | KT385642 |
| *G. rubicunda* 2 | b101/Tian/Taidong county, Taiwan, China | KT344058 | KT385553 | KT385640 |
| *G. rubicunda* 3 | b104/Tian/Taroko, Hualian, Taiwan, China | KT344059 | KT385554 | KT385641 |
| *G. rubicunda* 4 | 203/Chung/Baler Town, Philippines | KT344055 | **-** | **-** |
| *G. rubicunda* 5 | 556/Chung/Wulai, Taibei, Taiwan, China | KT344057 | **-** | **-** |
| *G. rubicunda* 6 | 23/Chung/Cibodas, Indonesia | KT344056 | **-** | **-** |
| *Goodyera schlechtendaliana* Rchb. f. 1 | a14/Tian/Pailong, Linzhi county, Xizang, China | KT344065 | KT385556 | KT385643 |
| *G. schlechtendaliana* 2 | b106/Tian/Mt. Lala Mountain, Taoyuan, Taiwan, China | KT344066 | KT385557 | KT385644 |
| *G. schlechtendaliana* 3 | b85/Tian/Linzhi County, Xizang, China | KT344067 | KT385558 | KT385645 |
| *G. schlechtendaliana* 4 | 399/Chung/Mt. Fansipan, Sapa, Viet Nam | KT344061 | **-** | **-** |
| *G. schlechtendaliana* 5 | 500/Chung/Mt. Fansipan, Sapa, Viet Nam | KT344062 | **-** | **-** |
| *G. schlechtendaliana* 6 | 627/Chung/Simakusi, Xinzhu County, Taiwan, China | KT344064 | **-** | **-** |
| *G. schlechtendaliana* 7 | 591/Chung/Mt. Fansipan, Sapa, Viet Nam | KT344063 | **-** | **-** |
| *G. schlechtendaliana* 8 | **-** | HM021568 | **-** | **-** |
| *G. schlechtendaliana* 9 | **-** | AF366897 | **-** | **-** |
| *Goodyera seikoomontana* Yamam. 1 | a22/Tian/Nanling, Guangdong, China | KT344069 | KT385559 | KT385646 |
| *G. seikoomontana* 2 | 109/Chung/Datong tribe, Yilan County, Taiwan, China | KT344068 | KT385560 | KT385647 |
| *Goodyera tesselata* Lodd. | **-** | HM141006 | **-** | **-** |
| *Goodyera thailandica* Seidenf. | b77/Ye/Simao, Yunnan, China | KT344070 | KT385561 | KT385648 |
| *Goodyera velutina* Maxim. ex Regel 1 | b04/Hu/Tianmu, Zhejiang, China | KT344073 | KT385562 | KT385649 |
| *G. velutina* 2 | b31/Hu & Li/Sanjiang Ecological Tourist Park, Sichuan, China | KT344075 | KT385564 | KT385651 |
| *G. velutina* 3 | b51/Tian & Dong /Wuzhishan, Hainan, China | KT344076 | KT385565 | KT385652 |
| *G. velutina* 4 | b55/Tian & Dong/Wuzhishan, Hainan, China | KT344077 | KT385566 | KT385653 |
| *G. velutina* 5 | b105/Tian/Mt. Lala, Taoyuan County, Taiwan, China | KT344074 | KT385563 | KT385650 |
| *G. velutina* 6 | 193/Chung/Mt. Shibi, Yunlin County, Taiwan, China | KT344071 | **-** | **-** |
| *G. velutina* 7 | 389/Chung/Mt. Fansipan, Sapa, Viet Nam | KT344072 | **-** | **-** |
| *G. velutina* 8 | **-** | HM140994 | **-** | **-** |
| *Goodyera viridiflora* (Blume) Blume 1 | a19/Tian/Nanling, Guangdong, China | KT344082 | KT385568 | KT385655 |
| *G. viridiflora* 2 | b19/Hu/Yezhutang, Malipo, Yunnan, China | KT344084 | KT385570 | KT385657 |
| *G. viridiflora* 3 | b24/Hu/Simao, Yunnan, China | KT344085 | KT385571 | KT385658 |
| *G. viridiflora* 4 | b107/Chung/Philippines | KT344083 | KT385569 | KT385656 |
| *G. viridiflora* 5 | 524/Chung/Simakusi, Xinzhui County, Taiwan, China | KT344080 | KT385567 | KT385654 |
| *G. viridiflora* 6 | 502/Chung/Mount Banahaw, Philippines | KT344079 | **-** | **-** |
| *G. viridiflora* 7 | 444/Chung/Cibodas, Indonesia | KT344078 | **-** | **-** |
| *G. viridiflora* 8 | 555/Chung/Halimun, Indonesia | KT344081 | **-** | **-** |
| *Goodyera vittata* (Lindl. ) Benth. ex Hook. f. | 706/Tian & Hu/Tongbiguan, Yingjiang, Yunnan, China | KT344086 | KT385572 | KT385659 |
| *Goodyera wolongensis* K. Y. Lang | c13/Tang/Wanglang, Sichuan, China | KT344087 | KT385573 | KT385660 |
| *Goodyera yamiana* Fukuy. | 78/Chung/Lanyu village, Taidong, Taiwan, China | KT344088 | KT385574 | KT385661 |
| *Goodyera pusilla* Blume 1 | a20/Tian/Wuzhishan, Ruyuan, Guangdong, China | KT344046 | KT385545 | KT385632 |
| *G. pusilla* 2 | b100/Tian/Mt. Doulan, Taitong, Taiwan, China | KT344047 | KT385546 | KT385633 |
| *Goodyera yunnanensis* Schltr. 1 | a13-2/Tian/Gangxiang, Bomi, Xizang, China | KT344089 | KT385575 | KT385662 |
| *G. yunnanensis* 2 | a13-3/Tian/Gangxiang, Bomi, Xizang, China | KT344090 | KT385576 | KT385663 |
| *Hayata merrillii* (Ames & Quisumb. ) T. C. Hsu & S. W. Chung | 542/Chung/Shuanglong, Nantou County, Taiwan, China | KT344080 | **-** | **-** |
| *Hayata tabiyahaensis* (Hayata) Aver. | 105/Chung/Mt. Qingshui, Hualian County, Taiwan, China | KT344092 | **-** | **-** |
| *Hetaeria affinis* (Griff. ) Seidenf. & Ormerod | 410/Chung/Tam Dao, Viet Nam | KT344093 | **-** | **-** |
| *Hetaeria youngsayei* Ormerod | 445/Chung/Bu Gia Map, Viet Nam | KT344095 | **-** | **-** |
| *Hetaeria oblongifolia* Blume* | 91/Chung/Taiwan，Chinia | KT344094 | KT385577 | JN166029 |
| *Hylophila lanceolata* (Blume) Miq. 1* | 475/Chung/Mt. Banahaw de Lucban, Philippines | KT344096 | KT385578 | JN166030 |
| *H. lanceolata* 2 | 169/Chung/Baler, Philippines | KT344097 | **-** | **-** |
| *H. lanceolata* 3 | **-** | JN166070 | **-** | **-** |
| *Hylophila nipponica* (Fukuy. ) T. P. Lin | 446/Chung/Lanyu village, Taidong, Taiwan, China | KT344098 | **-** | **-** |
| *Kreodanthus simplex* (C. Schweinf. ) Garay | **-** | FJ473328 | **-** | **-** |
| *Kuhlhasseltia integra* (Fukuy. ) T. C. Hsu & S. W. Chung | 83/Chung/Lanyu village, Taidong, Taiwan, China | KT344099 | **-** | **-** |
| *Kuhlhasseltia yakushimensis* (Yamam. ) Ormerod | 447/Chung/Jianshi Country, Xinzhu County, Taiwan, China | KT344100 | **-** | **-** |
| *Lepidogyne longifolia* (Blume) Blume 1* | 557/Chung/Mt. Banahaw de Lucban, Philippines | KT344101 | KT385579 | JN166032 |
| *L. longifolia 2* | **-** | JN166072 | **-** | **-** |
| *Ludisia discolor* (Ker Gawl. ) A. Rich. * | 448/Chung/Yinggeling, Hainan, China | KT344102 | KT385580 | AJ543911 |
| *Macodes dendrophila* Schltr. | 449/Chung/Baler, Philippines | KT344103 | **-** | **-** |
| *Macodes petola* (Blume) Lindl. | 450/Chung/Cibodas, Indonesia | KT344104 | **-** | **-** |
| *Microchilus arietinus* (Rchb. f. & warm) Ormerod | - | FJ473320 | - | - |
| *Myrmechis philippinensis* Ames | 512/Chung/Mt. Banahaw de Lucban, Philippines | KT344105 | **-** | **-** |
| *Myrmechis pumila* (Hook. f. ) Tang & F. T. Wang | 396/Chung/Mt. Fansipan, Viet Nam | KT344106 | **-** | **-** |
| *Odontochilus elwesii* C. B. Clarke ex Hook. f. | 400/Chung/Tam Dao, Viet Nam | KT344107 | **-** | **-** |
| *Odontochilus lanceolatus* (Lindl. ) Blume | 451/Chung/Yingziling, Yilan county, Taiwan, China | KT344108 | **-** | **-** |
| *Orchipedum wenzelii* (Ames) J. J. Sm. | 453/Chung/Baler, Philippines | KT344109 | **-** | **-** |
| *Pachyplectron arifolium* Schltr. 1 * | **-** | FJ473334 | FJ571286 | AJ310051 |
| *P. arifolium* 2 | **-** | AF348049 | **-** | **-** |
| *Platythelys querceticola* (Lindl. ) Garay * | - | FJ473336 | FJ571288 | AY368386 |
| *Pristiglottis montana* (Schltr. ) Cretz. & J. J. Sm. 1* | **-** | FJ473344 | AJ409444 | GQ917041 |
| *P. montana* 2 | **-** | JQ045488 | **-** | **-** |
| *Pterostylis longifolia* R. Br. * | **-** | AY134639 | AJ409445 | AJ310062 |
| *Rhomboda abbreviate* (Lindl. ) Ormerod | 413/Chung/Yinggeling, Hainan, China | KT344110 | **-** | **-** |
| *Rhomboda petelotii* (Gagnep. ) Ormerod | 406/Chung/Mt. Fansipan, Viet Nam | KT344111 | **-** | **-** |
| *Vrydagzynea albida* (Blume) Blume | 138/Chung/Baler, Philippines | KT344112 | **-** | **-** |
| *Vrydagzynea weberi* Ames | 144/Chung/Baler, Philippines | KT344113 | **-** | **-** |
| *Zeuxine strateumatica* (L. ) Schltr. * | 425/Chung/YanchaoCountry, Gaoxiong, Taiwan, China | KT344117 | KT385582 | AJ310080 |
| *Zeuxine affinis* (Lindl. ) Benth. ex Hook. f. | 518/Chung/Hengchun, Pingdong County, Taiwan, China | KT344114 | **-** | **-** |
| *Zeuxine odorata* Fukuy. | 85/Chung/Lanyu village, Taidong, Taiwan, China | KT344115 | **-** | **-** |
| *Zeuxine vieillardii* (Rchb. f. ) Schltr. | **-** |  | AJ409459 | AJ310081 |
| *Zeuxine* sp. | b103/Tian/Lanyu village, Taidong, Taiwan, China | KT344116 | KT385581 | KT385664 |

Abbreviations used: Chung, Chung Shih wen; Dong, Dong Quanying; Ge, Ge Bingjie. Hu, Hu Chao; Jiang, Jiang Hong; Li, Li Xinglin; Tang, Tang Ying; Tian, Tian Huaizhen; Wei, Wei Yumei; Ye, Ye Deping;

*represent different sample sequences combined together in combined data analysis.
