# Supplementary material for: Phylogenetic Analysis of a ‘Jewel Orchid’ Genus Goodyera (Orchidaceae) Based on DNA Sequence Data from Nuclear and Plastid Regions
Source: PLoS One. 2016 Feb 29;11(2):e0150366. doi: 10.1371/journal.pone.0150366 (PMC4771202; doi:10.1371/journal.pone.0150366)
Supplement: S2 Table — (DOCX) [file pone.0150366.s004.docx]

**Table. S2 Primers about sequences amplication and sequencing**

| Sequence | Primers（5’-3’） | Reference | Amplication protocol |
| --- | --- | --- | --- |
| ITS  *trn*L-F | 18S dir: CGTAACAAGGTTTCCGTAGG | Venora et al., 2000 ^[1]^ | 92°C 3min; 95°C 30s, 50°C 30s, 72°C 1min, 30cycles; 72°C 7min  94°C 2min; 94°C 1min, 50°C 30s, 72°C 1min, 30cycles; 72°C 7min |
|  | ITS4: TCCTCCGCTTATTGATATGC | White et al., 1990 ^[2]^ |  |
|  | c: CGAAATCGGTAGACGCTACG  d: GGGGATAGAGGGACTTGAAC  e: GGTTCAAGTCCCTCTATCCC | Taberlet et al., 1991 ^[3]^  Taberlet et al., 1991 ^[3]^  Taberlet et al., 1991 ^[3]^ |  |
| *mat*K 1  *mat*K 2 | f: ATTTGAACTGGTGACACGAG | Taberlet et al., 1991 ^[3]^ | 80°C 5min; 95°C 1min, 51°C 1min, 65°C 4min, 30cycles; 65°C 5min |
|  | 19F: CGTTCTGACCATATTGCACTATG  834R: AAAGACTCCARAAGATRTTG | Molvray et al., 2000 ^[4]^  Kocyan et al., 2004 ^[5]^ |  |
|  | 731F :TCTGGAGTCTTTCTTGAGCGA  580F: ACTAATACCCYATCCCATMC  trnK2R: AACTAGTCGGATGGAGTAG) | Gervendeel et al., 2001 ^[6]^  Kocyan et al., 2004 ^[5]^  Johnson & Soltis, 1994 ^[7]^ |  |

1. Venora G, Blangiforti S, Frediani M, Maggini E, Gelati MT. Nulclear DNA contents, rDNAs, chromatin organization, and karyotype evolution in *Vicia* sect. *faba*. Protoplasma. 2000; 213: 118-125.
2. White TJ, Bruns T, Lee S, Taylor JW. Ampliﬁcation and direct sequencing of fungal ribosomal RNA genes for phylogenetics, In Innis MA, Gelfand DH, Sninsky JJ, White TJ. PCR Protocols: A guide to methods and applications, San Diego: Academic Press; 1990, pp. 315-322.
3. Taberlet P, Gielly L, Pautou G, Bouvet J. Universal primers for amplification of three non-coding regions of chloroplast DNA. Plant Mol Biol. 1991; 17: 1105-1110.
4. Molvray M, Kores PJ, Chase MW. Polyphyly of mycoheterotrophic orchids and functional influences on floral and molecular characters. In: Wilson KL, Morrison DA, Monocots: Systematics and Evolution. Collingwood: CSIRO; 2000. pp. 441-448.
5. Kocyan A, Qiu YL, Endress PK, Conti EA. Phylogenetic analysis of Apostasioideae (Orchidaceae) based on ITS, *trnL-F* and *matK* sequences. Plant Syst Evol. 2004; 247: 203-213.
6. Gravendeel B, Chase MW, Vogel de EF, [Roos](https://sslvpn.ntu.edu.tw/,DanaInfo=www.amjbot.org+search?author1=Marco+C.+Roos&sortspec=date&submit=Submit) MC, [Mes](https://sslvpn.ntu.edu.tw/,DanaInfo=www.amjbot.org+search?author1=Ted+H.+M.+Mes&sortspec=date&submit=Submit), THM, Bachmann K. Molecular phylogeny of *Coelogyne* (Epidendroideae; Orchidaceae) based on plastid RFLPS, *matK*, and nuclear ribosomal ITS sequences: evidence for polyphyly. Amer J Bot. 2001; 88(10): 1915-1927.
7. Johnson LA, Soltis DE. *MatK* DNA sequences and phylogenetic reconstruction in Saxifragranceae s. str. Syst Bot. 1994; 19: 143-156.
